# The clinical significance of IL-6 s and IL-27 s in Bronchoalveolar lavage fluids from children with mycoplasma pneumoniae pneumonia

**DOI:** 10.1186/s12879-020-05017-3

**Published:** 2020-05-11

**Authors:** Jie Zhao, Yuyun Li, Wen Zhang

**Affiliations:** 1 Pediatrics, Zibo Central Hospital, Zibo, 255036 Shandong Province China; 2 Allergy Clinic, Zibo Central Hospital, Zibo, 255036 Shandong Province China; 3 Clinical Laboratory, Zibo Central Hospital, No. 54, Gongqingtuanxi Street, Zibo, 255036 Shandong Province China

**Keywords:** *Mycoplasma pneumoniae* pneumonia, Bronchoalveolar lavage fluids, IL-6, IL-27, Community-acquired pneumonia

## Abstract

**Background:**

IL-6 was associated with the severity of *mycoplasma pneumoniae* pneumonia (MPP). But the relationship between IL-27 and MPP was unknown.

**Methods:**

Ninety-eight patients with MPP < 14 years old were enrolled in this study and divided into groups by severity (mild cases and severe cases), infection types (*MP* single infection group and *MP* mixed infection group) and DNA loads (low *MP* DNA loads group and high *MP* DNA loads group), respectively. Fifteen children with foreign bodies in bronchus were also enrolled as control. IL-6 s and IL-27 s in bronchoalveolar lavage fluids (BALFs) from these children were measured by ELISA.

**Results:**

There were significant differences in IL-6 s of BALFs from patients between mild cases and severe cases, *MP* single infection group and *MP* mixed infection group, and low *MP* DNA loads group and high *MP* DNA loads group, respectively (*P* < 0.05). Compared with IL-6 s of BALFs from control, IL-6 s in BALFs from the 6 patient groups were significantly higher (*P* < 0.05). IL-27 s in BALFs from *MP* mixed infection group were significantly lower than those from *MP* single infection group and control (*P* < 0.05) respectively.

**Conclusion:**

IL-6 was firmly associated with MPP and had potential application in clinical practice while IL-27 was not related to *MP* infection.

## Background

*Mycoplasma pneumoniae* (MP) is one of the main pathogens in respiratory infections in children. It causes more than 40% of community-acquired pneumonia (CAP) cases in children, of which 18% cases need hospitalization [1]. At present there are many problems with patients suffering from *Mycoplasma pneumoniae* pneumonia (MPP) such as the increasing macrolide resistance rate [2], the complex multiple systemic complications [3], the increasing occurrence of refractory MPP [4]. Therefore, MPP has attracted great attentions from many practitioners and patients. Immune function disorders are involved in the pathogenesis of MPP [5]. And IL-6 plays important role in regulating immune functions [6]. It is involved in the infection process of *MP* and plays an important role in the pathogenesis of MPP [7]. One study suggests that IL-6 is associated with the severity of MPP [8]. IL-27 is another important cytokine which is firmly associated with IL-6. It can induce the secretion of IL-6 [9] and can also block the activity of IL-6 by its subunit of IL-27 p28 [10], which plays dual roles of pro-inflammation and anti-inflammation. Plfans et al [11] reports that IL27 is positively associated with IL6 in patients with brain injury. However, no reports have been found about whether there is any relationship between them in MPP.

Fiberoptic bronchoscopy and bronchoalveolar lavage is safe and effective in the diagnosis and treatment of MPP, which can provide bronchoalveolar lavage fluids (BALFs) for research. BALFs can reflect the pathological and biochemical changes of lung tissues directly. However, there have been few reports about IL-6 s and IL-27 s in BALFs from MPP patients. Only a few reports about the relationship between IL-6 in sera and the severity of MPP [8] can be found.

In this study, the levels of IL-6 s and IL-27 s in BALFs from MPP patients and control were measured to explore their clinical significances.

## Methods

### Including, excluding, and grouping criteria

In this study, the diagnosis of MPP met the following criteria: 1) fever, coughing, and other respiratory tract infection symptoms; 2) chest radiographic examinations with bronchial pneumonia, interstitial pneumonia, segmental or lobar pneumonia, and even pleural effusion; and 3) a single serum anti- *MP* IgM antibody titer of ≥1:160 at the acute phase following admission (in those with no history of respiratory infections in the past 3 months) and a positive PCR test for *MP*. Patients with any of the following criteria [12, 13] would be diagnosed as severe cases: 1) tachypnea or tachycardia (Tachypnea was defined as a respiratory rate of > 40/m for children aged 1–5 years, and 30/m for children aged> 5 years. Tachycardia was defined as > 140 bpm for children aged 1–3 years, > 120 bpm for children aged 3–5, > 118 bpm for children aged 5–10 years, and > 100 bpm for children aged > 10 years of age.) with or without nasal flare, moaning, three concave sign, and cyanosis; 2) hypoxemia (SaO2 ≤ 92%); 3) refractory MPP; 4) multilobar involvement or involvement area ≥ 2/3 on chest radiographs; 5) pleural effusion (> 300 ml), severe atelectasis, pulmonary necrosis and pulmonary abscess. 6) Other severe complications (central nervous system infections, heart failure, myocarditis, gastrointestinal hemorrhage, and obvious electrolyte/ acid-base balance disorders). Patients having infections within 3 months, or suffering from known coexisting chronic, progressive or oncological illnesses, or receiving corticosteroids or immunosuppressive agents within 3 months, or with immune hypofunctions or immune related diseases, or allergic diseases or suspected allergic diseases (including allergic rhinitis and atopic dermatitis) or asthmas were excluded from the research.

All the patients were divided into severe cases and mild cases by the severity of the diseases. The whole patients could also be divided into *MP* single infection group and *MP* mixed infection group according to the infection types as well as low *MP* DNA loads (<10^5^copies/ml) and high *MP* DNA loads (≥10^5^copies/ml) according to the MP DNA loads.

### Data collections

Data including ages, genders, clinical signs and symptoms, laboratory and radiological findings were collected from patients during Jan 1st and Dec 31st of the year 2017. All chest radiographs and computed tomography were reviewed by two experienced radiologists and they agreed on the conclusions.

### MP DNA extractions, detections and quantifications

MP DNAs from BALFs were extracted using QIAamp DNA MINI kit (Qiagen, Hilden Germany). The target gene for detecting MP by PCR was a segment of gene p1 adhesion with 150 bp (P1–178: CAATGCCATCAACCCGCGCTTAACC,P1–331: CGTGGTTTGTTGACTGCCACTGCCG). The PCR conditions were: 30 cycles of 94 °C for 30 s, 62 °C for 30 s and 72 °C for 30 s. MP DNA was quantified using Mycoplasma pneumoniae DNA Fluorescence Diagnostic Kit (Shengxiang Biotechnology Co. Ltd., Hunan Province, China) with ABI PRISM 7500 instrument (Applied Biosystems TM, Foster City, California, United States). The experiments were strictly conducted in accordance with the manufacturers’ instructions.

### Collections of BALFs

Bronchoscopy was performed within 3 days after hospital admission for patients with MPP and immediately after hospital admission to remove the foreign bodies in bronchus from children in control. Flexible fiber optic bronchoscopy and bronchoalveolar lavage were performed following the guidelines described previously [14]. BALFs were collected from these children and stored in − 80 °C freezer.

### The assays of IL-6 s and IL-27 s

IL-6 s and IL-27 s in BALFs from the children were measured by sandwich enzyme-linked immunosorbent assay (ELISA) with commercial reagent kits (Abcan Company, USA). The experiments were strictly conducted in accordance with the manufacturer’s instructions.

### Statistical analysis

Statistical analyses were performed using SPSS21.0 Statistical package. Continuous variables were reported as means ± standard deviations. ANOVA was used to compare means of multiple groups. LSD test was used for the inter-comparison of 2 means in the multiple groups. The ages and genders between MPP patients and control were compared with t test and x^2^ test, respectively. MP DNA loads, IL-6 s and IL-27 s were shown and compared by box plots. *P* < 0.05 was considered to indicate a statistically significant difference.

## Results

### General information

From Jan 1st to Dec 31st of the year 2017, a total of 98 hospitalized children with MPP according to the including criteria and excluding criteria were enrolled. All patients including 47 male patients and 51 female ones underwent fiberoptic bronchoscopy, and BALFs were collected from them. The ages of the patients ranged from 1 to 13 years (6.63 ± 2.56 years). During the study period, 15 children with foreign bodies in bronchus consisting of 8 males and 7 females were also enrolled as control. The ages of them were among 1 to 9 years (5.40 ± 2.27 years). There were no inflammatory changes on chest radiographs and computed tomography of children in control. No symptoms of respiratory infections including coughing, fever and sore throat existed after brochoscopy in control. WBCs and CRPs in sera of the control were in normal ranges. No significant differences in ages (t = 1.76, *p*>0.05) and sex ratio (x^2^ = 0.15, *p*>0.05) between the patients and control were observed.

Severe cases consisted of 26 male patients and 27 female ones. The ages of them were 6.91 ± 2.22 years old. Mild cases included 45 patients with 21 boys and 24 girls. The ages of them averaged 6.05 ± 2.73 years old. *MP* single infections (60 cases) consisted of 25 male patients and 35 female ones. The ages of them were 6.81 ± 2.67 years old. *MP* mixed infections included 38 patients with 22 boys and 16 girls. The ages of them averaged 6.34 ± 2.38 years old. Low *MP* DNA loads (1.0 × 10^3^copies/ml ≤ 50 cases<10^5^copies/ml) consisted of 23 male patients and 27 female ones. The ages of them were 6.92 ± 2.86 years old. High *MP* DNA loads (48 cases ≥10^5^copies/ml) included 48 patients with 24 boys and 24 girls. The ages of them averaged 5.86 ± 2.67 years old. No significant differences in ages (t = 1.72, *p*>0.05; t = 0.88, *p*>0.05;t = 1.89, *p*>0.05) and sex ratio (x^2^ = 0.06, *p*>0.05; x^2^ = 2.45, *p*>0.05; x^2^ = 0.16, *p*>0.05) were observed between severe cases and mild cases, *MP* single infections and *MP* mixed infections as well as low *MP* DNA loads and high *MP* DNA loads.

### MP DNA loads

**Fig. 1:**
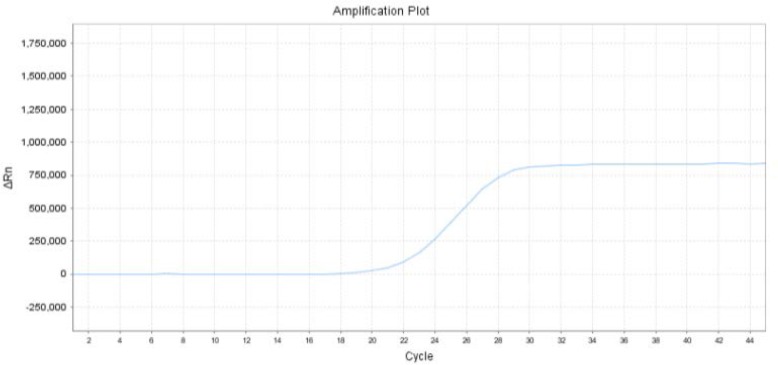
The amplification plot of one sample (2.9*10^7^copies/ml)

**Fig. 2:**
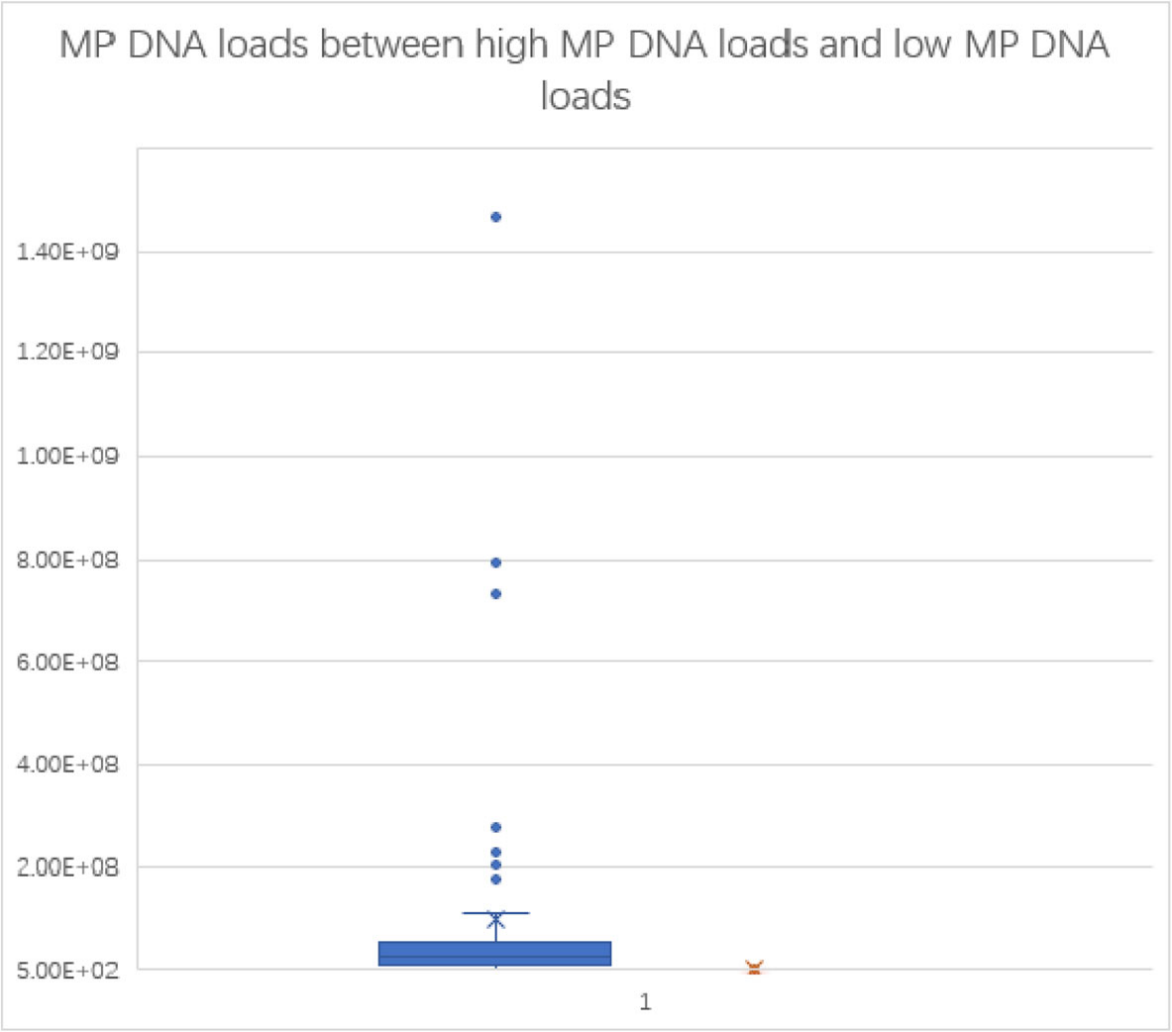
Box plot of MP DNA loads

After confirmation by PCR, all the samples from BALFs were quantified by quantitive real-time PCR with MP DNA loads ranging from 2.98 × 10^3^ copies/ml to 1.47 × 10^9^ copies/ml. (Figs. 1, 2).

### The comparison of IL-6 s and IL-27 s in BALFs among severe cases, mild cases and control, respectively

**Fig. 3:**
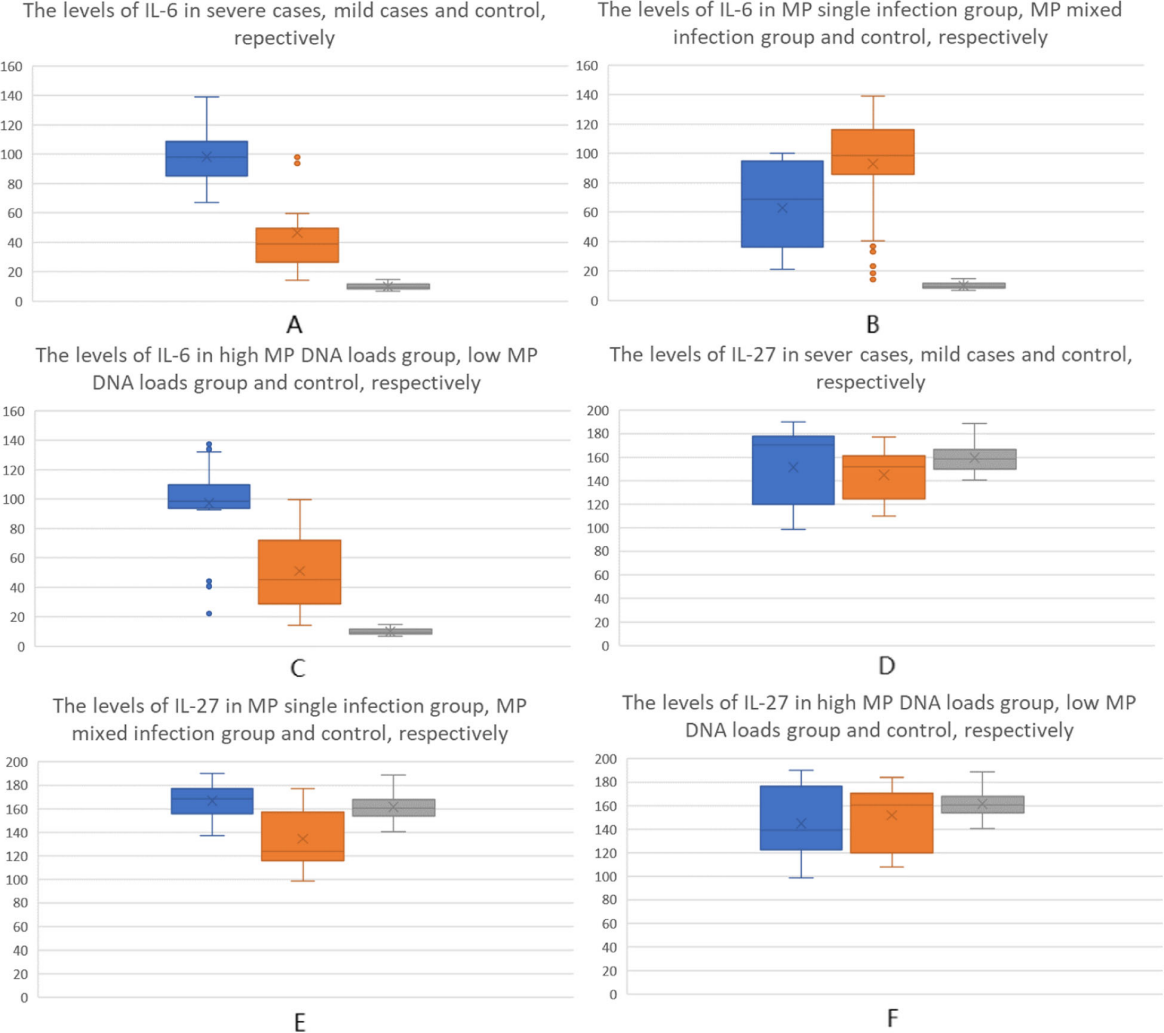
Box plot of IL-6 s and IL-27 s

**Fig. 4:**
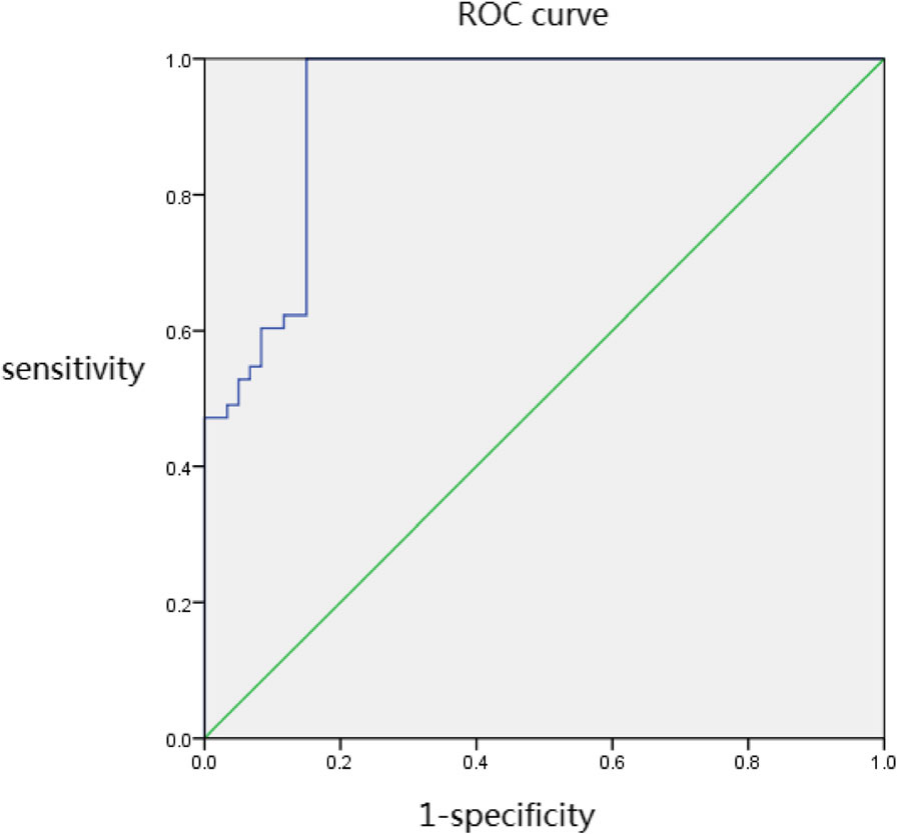
ROC curve analysis of IL-6 s in mild case and severe cases of MPP

IL-6 s in BALFs from severe cases were higher than those from control and mild cases. There were significant differences between them (*p* < 0.05). IL-6 s in BALFs from mild cases were significantly higher than those from control (*p* < 0.05) (Fig. 3 A). It suggested that IL-6 s in BALFs were firmly associated with the severity of the disease. By ROC curve analysis (Fig. 4), the cut off value of IL-6 was 63.055 pg/L. The sensitivity and specificity were 98.10 and 85.00%, respectively. IL-27 s in BALFs from severe cases and mild cases were slightly decreased than those from control, but there were no significant differences in IL-27 s among them (*p* > 0.05). (Table 1) (Fig. 3d).

**Table 1:** The comparison of IL-6 s and IL-27 s in BALFs among three groups of children respectively

Groups	cases	IL-6 s (pg/ml)	IL-27 s (pg/ml)
MPP severe cases	53	98.13 ± 21.01^a b^	151.40 ± 26.77
MPP mild cases	45	46.62 ± 26.68^a^	144.75 ± 18.77
control F value P	15	10.06 ± 2.26 119.98 0.00	161.62 ± 13.35 2.74 0.07

“^a^” signified that there was significant difference between MPP cases and control. “^b^” signified that there was significant difference between MPP severe cases and MPP mild cases

### The comparison of IL-6 s and IL-27 s in BALFs among *MP* single infection group, *MP* mixed infection group and control, respectively

IL-6 s in BALFs from *MP* mixed infection group were higher than those from control and *MP* single infection group. There were significant differences between them (*p* < 0.05). IL-6 s in BALFs from *MP* single infection group were significantly higher than those from control (*p* < 0.05) (Fig. 3b). It suggested that IL-6 s in BALFs were associated with MP mixed infections. IL-27 s in BALFs from *MP* mixed infection group were significantly lower than those from control and *MP* single infection group (*p* < 0.05), which suggested that IL-27 was negatively associated with other pathogens but *MP*. But no significant differences were found in IL-27 s from BALFs between *MP* single infection group and control (*p* > 0.05). (Table 2) (Fig. 3e).

**Table 2:** The comparison of IL-6 s and IL-27 s in BALFs among three groups of children respectively

Groups	cases	IL-6 s (pg/ml)	IL-27 s (pg/ml)
MP single infection	60	62.82 ± 28.52^a^	166.77 ± 13.23
MP mixed infection	38	92.89 ± 36.70^a b^	119.26 ± 9.37^a b^
control F value P	15	10.06 ± 2.26 42.14 0.00	161.62 ± 13.35 187.57 0.00

“^a^” signified that there was significant difference between MPP cases and control. “^b^” signified that there was significant difference between MPP mixed infection group and MPP single infection group

### The comparison of IL-6 s and IL-27 s in BALFs among high *MP* DNA loads group, low *MP* DNA loads group and control, respectively

IL-6 s in BALFs from high *MP* DNA loads group were higher than those from control and low *MP* DNA loads group. There were significant differences between them (*p* < 0.05) (Fig. 3c). The levels of IL-6 in BALFs from low *MP* DNA loads group were also significantly higher than those from control (*p* < 0.05). There were no significant differences in IL-27 s in BALFs from high *MP* DNA loads group, low *MP* DNA loads group and control (*p* > 0.05). (Table 3) (Fig. 3f).

**Table 3:** The comparison of IL-6 s and IL-27 s in BALFs among three groups of children respectively

groups	cases	IL-6 s(pg/ml)	IL-27 s(pg/ml)
high MP DNA loads	50	101.71 ± 11.31^a b^	144.90 ± 27.74
low MP DNA loads	48	41.26 ± 5.47^a^	151.93 ± 24.04
control F value P	15	10.06 ± 2.26 87.48 0.00	161.62 ± 13.35 2.86 0.06

“^a^” signified that there was significant difference between MP DNA loads and control. “^b^” signified that there was significant difference between high MP DNA loads group and low MP DNA loads group

## Discussions

MPP is a common respiratory infection in children and a leading cause of death in China [15]. In recent years, patients infected by *MP* have increased year by year in the worldwide [16]. So MPP has drawn much attention from medical practitioners. The main clinical manifestations include fever, coughing, shortness of breath and continuous dry and wet rales in the lung. Patients often present from mild to severe symptoms. It is reported that IL-6 is associated with the severity of MPP [8]. But no reports about IL-6 relating to MP DNA loads and MP infection types in patients with MPP have been found. There are also no reports found about the relationship between IL-27 and MPP. So the research aimed to explore the relationship of IL-6 and MPP further as well as IL-27 and MPP.

IL-6 is an important cytokine that has dual functions in the process of inflammation [6]. It is mainly secreted by Th2 cells and can promote the secretion of protective antibodies to extracellular microbial pathogens [6]. IL-6 plays an important role in the pathogenesis of MPP [7]. Our previous research suggested that IL-6-174 G/C genotype increased *MP* infection in patients [17], which also suggested that IL-6 was closely related to *MP* infection. In the research, IL-6 s in BALFs were significantly higher in patients with MPP than those in control. There were significant differences in IL-6 s between severe cases and mild cases. It suggested that IL-6 was closely related to the severity of the disease, which was similar to the previous report [8]. IL-6 s in *MP* mixed infection group were significantly higher than those in *MP* single infection group, which suggested that IL-6 may be associated with other pathogens [18]. IL-6 was closely associated with *MP* DNA loads for there were significant differences in IL-6 s of BALFs between high *MP* DNA loads group and low *MP* DNA loads group. These results had not been found reported previously. Additionally, IL-6 s in RMPP patients were significant higher than those in General MPP patients [19]. IL-6 was also associated with pleural effusion [20] and radiological appearance [8] in MPP patients. Guo L et al. reported that IL-6 s in serum of patients infected by macrolide-resistant strains were higher than those infected by non macrolide-resistant strains [21]. Therefore IL-6 was closely related to MPP**.** In the future, it may be used as an indicator for reflecting the severity and the infection state of the disease.

IL-27 is produced by antigen-presenting cells upon exposure to microbial-derived molecules and inflammatory stimuli [22]. It has emerged as a pro-inflammatory factor that signals via binding to IL-27R, which consists of IL-27Ra (WSX-1/TCCR) and glycoprotein 130 subunits, and mediates various inflammation-promoting biological activities involved in the pathogenesis of many inflammation-related diseases [23]. Increasing evidence suggests that IL-27 is a strong inducer of chemokines and pro-inflammatory cytokines including IL-6 by activated neutrophils, monocytes, and macrophages [9]. IL-27 is a heterodimeric cytokine constituted of two subunits, EBI3 and IL-27-p28 [11], and IL-27 p28 can block the activity of IL-6 [10]. Therefore, IL-27 may maintain the level of IL-6 in a balance state.

In this research, there were no significant differences in the levels of IL-27 between MPP groups and control, *MP* severe cases and *MP* mild cases, high *MP* DNA loads and low *MP* DNA loads, which suggested that IL-27 was not related to *MP* infection. However, the levels of IL-27 in *MP* mixed infection group were significantly lower than those in *MP* single infection group and control, which suggested that IL-27 may be negatively related with other pathogens. Further research could be conducted with the increasing pneumonias infected by other pathogens in our hospital. However, it was postulated that IL-27 was not involved in the pathogenesis of MPP.

The research still had some limitations. The samples size included in the study was not large enough. The pathogens in the *MP* mixed infection group included many kinds of pathogens such as *respiratory syncytial virus*, *chlamydia pneumonia*, and *influenza A virus*. The number of each kind of pathogens co-infected in the *MP* mixed infection group was too few to get a statistical analysis. Therefore, the association of IL-6, IL-27 and other pathogens was hard to analyze.

## Conclusion

In summary, IL-6 was closely related with the severity, *MP* DNA loads, and *MP* mixed infections in the patients with MPP. It was a potential indicator in clinical practice. However, IL-27 was not related to *MP* infections but may be related to other pathogens. Both of them should be studied further.

## Data Availability

The datasets used and/or analyzed during the current study are available from the corresponding author on reasonable request.

## Abbreviations

MPP: *mycoplasma pneumoniae* pneumonia
*MP*: *mycoplasma pneumoniae*
BALFs: bronchoalveolar lavage fluids
CAP: community-acquired pneumonia
